# 1-[4-(2-Amino­anilino)phen­yl]-2,2,2-trifluoro­ethanone

**DOI:** 10.1107/S1600536810008937

**Published:** 2010-04-24

**Authors:** Angelika Dorn, Dieter Schollmeyer, Stefan A. Laufer

**Affiliations:** a Institute of Pharmacy, Department of Pharmaceutical and Medicinal Chemistry, Eberhard-Karls-University Tübingen, Auf der Morgenstelle 8, 72076 Tübingen, Germany; b Department of Organic Chemistry, Johannes Gutenberg-University Mainz, Duesbergweg 10-14, 55099 Mainz, Germany

## Abstract

In the title compound, C_14_H_11_F_3_N_2_O, the two aromatic rings are oriented at a dihedral angle of 70.84 (8)°. The crystal structure displays inter­molecular N—H⋯O and N—H⋯F inter­actions.

## Related literature

For 2,2,2-trifluoroacetophenones as intermediates for further Buchwald–Hartwig coupling, see: Colard *et al.* (1994 ▶); Schenck *et al.* (2004 ▶).
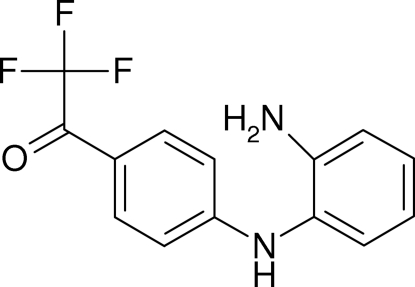


## Experimental

### 

#### Crystal data

C_14_H_11_F_3_N_2_O
*M* *_r_* = 280.25
Orthorhombic, 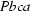

*a* = 13.0385 (13) Å
*b* = 8.7129 (7) Å
*c* = 22.424 (2) Å
*V* = 2547.4 (4) Å^3^
*Z* = 8
Mo *K*α radiation
μ = 0.12 mm^−1^
*T* = 173 K
0.4 × 0.3 × 0.2 mm

#### Data collection

Bruker SMART APEXII diffractometer
21134 measured reflections
3033 independent reflections
2498 reflections with *I* > 2σ(*I*)
*R*_int_ = 0.026

#### Refinement

*R*[*F* ^2^ > 2σ(*F* ^2^)] = 0.045
*wR*(*F* ^2^) = 0.127
*S* = 1.06
3033 reflections
181 parameters
H-atom parameters constrained
Δρ_max_ = 0.67 e Å^−3^
Δρ_min_ = −0.29 e Å^−3^

### 

Data collection: *APEX2* (Bruker, 2006 ▶); cell refinement: *SAINT* (Bruker, 2006 ▶); data reduction: *SAINT*; program(s) used to solve structure: *SIR97* (Altomare *et al.*, 1999 ▶); program(s) used to refine structure: *SHELXL97* (Sheldrick, 2008 ▶); molecular graphics: *PLATON* (Spek, 2009 ▶); software used to prepare material for publication: *PLATON*.

## Supplementary Material

Crystal structure: contains datablocks I, global. DOI: 10.1107/S1600536810008937/bt5212sup1.cif

Structure factors: contains datablocks I. DOI: 10.1107/S1600536810008937/bt5212Isup2.hkl

Additional supplementary materials:  crystallographic information; 3D view; checkCIF report

## Figures and Tables

**Table 1:** Hydrogen-bond geometry (Å, °)

*D*—H⋯*A*	*D*—H	H⋯*A*	*D*⋯*A*	*D*—H⋯*A*
N7—H7*B*⋯F19^i^	0.91	2.32	3.2077 (19)	166
N8—H8⋯O16^ii^	0.96	2.06	2.9757 (17)	159

Symmetry codes: (i) 
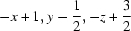
; (ii) 
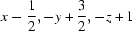
.

**Fig. 1.**
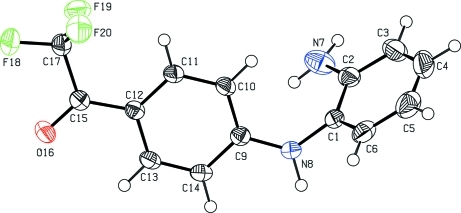
View of the title compound. Displacement ellipsoids are drawn at the 50% probability level.

## supplementary crystallographic information

### Comment

2,2,2-Trifluoroacetophenones are well known as building-blocks in the synthesis of therapeutic agents for example as acetylcholinesterase inhibitors (Colard *et al.*, 1994), as anticonvulsants (Schenck *et al.*, 2004) or as hPPAR agonists. The title compound was prepared in the course of our studies on acetophenone derivatives as potent p38 mitogen-activated protein (MAP) kinase inhibitors.

In the molecule (Fig. 1), rings A (C1—C6) and B (C10—C15) are, of course, planar and they are oriented at a dihedral angle of A/B = 70.84 (8)°. In the crystal structure, intermolecular N8—H8···O16 (2,06 Å) and N7—H7B···F19 (2,32 Å) interactions link the molecules into a three dimensional network. The N8—H8···O16 hydrogen bond forms a chain along the a-axis whereas the N7—H7B···F19 interaction connects two of this chains in direction parallel to the c-axis.

### Experimental

For the preparation of the title compound a mixture of 501 mg (2.4 mmol) 1-(4-chlorophenyl)-2,2,2-trifluoroethanone, 332 mg (2.4 mmol) 2-nitroaniline, 1400 mg (4.3 mmol) Cs_2_CO_3_,90 mg (0.19 mmol) 2-(dicyclohexylphosphino)-2'-, 4'-, 6'-triisopropylbiphenyl and 20 mg (0.09 mmol) Pd(OAc)_2_ in 2 ml absolute *tert-*butanol and 8 ml absolute toluol was stirred for 2 h at 90 °C under an atmosphere of argon. The mixture was diluted with water then extracted with ethyl acetate. The extracts were combined, washed with saturated saline solution, and then dried over Na_2_SO_4_. The solvent was removed under vacuum and the crude product was dissolved in 10 ml ethanol, 840 mg (3.71 mmol) tin(II)chloride-dihydrate was added and stirred for 5 h at 348 K. After cooling down to room temperature the mixture was quenched with 20 ml ice-water and after alkalization with NaOH (20 %) extracted three-times with ethyl acetate. The combined organic layer was washed twice with water, dried (Na_2_SO_4_), and evaporated under reduced pressure. The residue was purified by flashchromatography (SiO_2_ 60, hexane / ethyl acetate) (yield: 21.5 %). Crystals of the title compound were obtained by slow evaporation of a methanol *d6*solution at room temperature.

### Refinement

Hydrogen atoms attached to carbons were placed at calculated positions with C—H = 0.95 Å (aromatic) or 0.98–0.99 Å (*sp*^3^ C-atom). Hydrogen atoms attached to N7 and N8 were located in difference Fourier maps. All H atoms were refined in the riding-model approximation with isotropic displacement parameters  set at 1.2–1.5 times of the *U*_eq_ of the parent atom.

### Crystal data

**Table:**

C_14_H_11_F_3_N_2_O	*F*(000) = 1152
*M**_r_* = 280.25	*D*_x_ = 1.461 Mg m^−^^3^
Orthorhombic, *P**b**c**a*	Mo *K*α radiation, λ = 0.71073 Å
Hall symbol: -P 2ac 2ab	Cell parameters from 7908 reflections
*a* = 13.0385 (13) Å	θ = 2.4–27.7°
*b* = 8.7129 (7) Å	µ = 0.12 mm^−^^1^
*c* = 22.424 (2) Å	*T* = 173 K
*V* = 2547.4 (4) Å^3^	Block, brown
*Z* = 8	0.4 × 0.3 × 0.2 mm

### Data collection

**Table:**

Bruker SMART APEXII diffractometer	2498 reflections with *I* > 2σ(*I*)
Radiation source: sealed Tube	*R*_int_ = 0.026
graphite	θ_max_ = 27.9°, θ_min_ = 1.8°
CCD scan	*h* = −17→17
21134 measured reflections	*k* = −11→8
3033 independent reflections	*l* = −29→29

### Refinement

**Table:**

Refinement on *F*^2^	Primary atom site location: structure-invariant direct methods
Least-squares matrix: full	Secondary atom site location: difference Fourier map
*R*[*F*^2^ > 2σ(*F*^2^)] = 0.045	Hydrogen site location: inferred from neighbouring sites
*wR*(*F*^2^) = 0.127	H-atom parameters constrained
*S* = 1.06	*w* = 1/[σ^2^(*F*_o_^2^) + (0.0601*P*)^2^ + 1.4118*P*] where *P* = (*F*_o_^2^ + 2*F*_c_^2^)/3
3033 reflections	(Δ/σ)_max_ < 0.001
181 parameters	Δρ_max_ = 0.67 e Å^−^^3^
0 restraints	Δρ_min_ = −0.28 e Å^−^^3^

### Special details

**Table:**

Geometry. All esds (except the esd in the dihedral angle between two l.s. planes) are estimated using the full covariance matrix. The cell esds are taken into account individually in the estimation of esds in distances, angles and torsion angles; correlations between esds in cell parameters are only used when they are defined by crystal symmetry. An approximate (isotropic) treatment of cell esds is used for estimating esds involving l.s. planes.
Refinement. Refinement of *F*^2^ against ALL reflections. The weighted *R*-factor wR and goodness of fit *S* are based on *F*^2^, conventional *R*-factors *R* are based on *F*, with *F* set to zero for negative *F*^2^. The threshold expression of *F*^2^ > σ(*F*^2^) is used only for calculating *R*-factors(gt) etc. and is not relevant to the choice of reflections for refinement. *R*-factors based on *F*^2^ are statistically about twice as large as those based on *F*, and *R*- factors based on ALL data will be even larger.

### Fractional atomic coordinates and isotropic or equivalent isotropic displacement parameters (Å^2^)

**Table:**

	*x*	*y*	*z*	*U*_iso_*/*U*_eq_	
C1	0.18780 (11)	0.80106 (18)	0.63368 (7)	0.0277 (3)	
C2	0.19637 (13)	0.7530 (2)	0.69312 (7)	0.0330 (4)	
C3	0.11693 (16)	0.7953 (2)	0.73256 (8)	0.0479 (5)	
H3	0.1203	0.7641	0.7731	0.057*	
C4	0.03490 (16)	0.8806 (3)	0.71344 (11)	0.0580 (6)	
H4	−0.0181	0.9060	0.7408	0.070*	
C5	0.02813 (15)	0.9299 (3)	0.65535 (11)	0.0546 (6)	
H5	−0.0282	0.9908	0.6427	0.065*	
C6	0.10447 (13)	0.8895 (2)	0.61572 (8)	0.0381 (4)	
H6	0.1000	0.9226	0.5754	0.046*	
N7	0.27946 (14)	0.6728 (2)	0.71200 (7)	0.0486 (4)	
H7A	0.3249	0.6275	0.6871	0.073*	
H7B	0.2675	0.6308	0.7483	0.073*	
N8	0.26108 (10)	0.75201 (16)	0.59059 (5)	0.0291 (3)	
H8	0.2297	0.6912	0.5600	0.035*	
C9	0.35838 (11)	0.80793 (18)	0.58519 (6)	0.0255 (3)	
C10	0.39963 (12)	0.91396 (18)	0.62580 (6)	0.0271 (3)	
H10	0.3594	0.9488	0.6584	0.033*	
C11	0.49855 (12)	0.96782 (18)	0.61856 (6)	0.0272 (3)	
H11	0.5256	1.0394	0.6464	0.033*	
C12	0.55966 (11)	0.91856 (17)	0.57084 (6)	0.0253 (3)	
C13	0.51717 (12)	0.81270 (19)	0.53022 (6)	0.0284 (3)	
H13	0.5571	0.7789	0.4973	0.034*	
C14	0.41990 (12)	0.75778 (19)	0.53712 (6)	0.0288 (3)	
H14	0.3934	0.6854	0.5094	0.035*	
C15	0.66340 (11)	0.97035 (18)	0.55983 (6)	0.0266 (3)	
O16	0.71615 (9)	0.93617 (15)	0.51702 (5)	0.0362 (3)	
C17	0.71528 (12)	1.0765 (2)	0.60606 (7)	0.0322 (3)	
F18	0.81079 (8)	1.10979 (14)	0.59054 (5)	0.0480 (3)	
F19	0.72009 (9)	1.00909 (14)	0.65988 (4)	0.0457 (3)	
F20	0.66562 (9)	1.20895 (13)	0.61292 (6)	0.0490 (3)	

### Atomic displacement parameters (Å^2^)

**Table:**

	*U*^11^	*U*^22^	*U*^33^	*U*^12^	*U*^13^	*U*^23^
C1	0.0266 (7)	0.0279 (8)	0.0286 (7)	−0.0045 (6)	0.0007 (5)	−0.0050 (6)
C2	0.0395 (9)	0.0301 (8)	0.0294 (7)	−0.0087 (7)	0.0016 (6)	−0.0028 (6)
C3	0.0580 (12)	0.0506 (12)	0.0350 (9)	−0.0185 (9)	0.0134 (8)	−0.0102 (8)
C4	0.0368 (10)	0.0683 (15)	0.0690 (14)	−0.0087 (10)	0.0175 (9)	−0.0283 (12)
C5	0.0323 (9)	0.0582 (13)	0.0731 (15)	0.0060 (9)	−0.0043 (9)	−0.0257 (11)
C6	0.0323 (8)	0.0357 (9)	0.0461 (9)	−0.0009 (7)	−0.0083 (7)	−0.0099 (7)
N7	0.0645 (11)	0.0484 (10)	0.0328 (7)	0.0107 (8)	−0.0015 (7)	0.0043 (7)
N8	0.0285 (6)	0.0334 (7)	0.0254 (6)	−0.0031 (5)	−0.0012 (5)	−0.0067 (5)
C9	0.0268 (7)	0.0262 (7)	0.0235 (6)	0.0018 (6)	−0.0019 (5)	0.0023 (5)
C10	0.0291 (7)	0.0277 (8)	0.0246 (6)	0.0013 (6)	0.0031 (5)	−0.0041 (6)
C11	0.0300 (7)	0.0265 (8)	0.0252 (7)	−0.0009 (6)	0.0008 (5)	−0.0049 (6)
C12	0.0270 (7)	0.0265 (8)	0.0225 (6)	0.0027 (6)	0.0004 (5)	0.0005 (5)
C13	0.0315 (7)	0.0329 (8)	0.0208 (6)	0.0051 (6)	0.0011 (5)	−0.0029 (6)
C14	0.0334 (7)	0.0309 (8)	0.0221 (6)	0.0005 (6)	−0.0032 (6)	−0.0047 (6)
C15	0.0277 (7)	0.0272 (8)	0.0250 (7)	0.0041 (6)	0.0003 (5)	0.0019 (6)
O16	0.0313 (6)	0.0471 (7)	0.0302 (6)	0.0038 (5)	0.0073 (4)	−0.0043 (5)
C17	0.0301 (8)	0.0333 (9)	0.0331 (8)	−0.0041 (6)	0.0044 (6)	−0.0016 (6)
F18	0.0326 (5)	0.0561 (7)	0.0552 (7)	−0.0143 (5)	0.0085 (5)	−0.0073 (5)
F19	0.0530 (7)	0.0552 (7)	0.0289 (5)	−0.0147 (5)	−0.0056 (4)	−0.0012 (4)
F20	0.0485 (6)	0.0319 (6)	0.0667 (7)	−0.0023 (5)	0.0089 (5)	−0.0128 (5)

### Geometric parameters (Å, °)

**Table:**

C1—C6	1.391 (2)	C9—C10	1.404 (2)
C1—C2	1.402 (2)	C9—C14	1.413 (2)
C1—N8	1.4244 (19)	C10—C11	1.382 (2)
C2—N7	1.357 (2)	C10—H10	0.9500
C2—C3	1.411 (2)	C11—C12	1.402 (2)
C3—C4	1.371 (3)	C11—H11	0.9500
C3—H3	0.9500	C12—C13	1.410 (2)
C4—C5	1.374 (4)	C12—C15	1.447 (2)
C4—H4	0.9500	C13—C14	1.364 (2)
C5—C6	1.380 (3)	C13—H13	0.9500
C5—H5	0.9500	C14—H14	0.9500
C6—H6	0.9500	C15—O16	1.2180 (18)
N7—H7A	0.9044	C15—C17	1.545 (2)
N7—H7B	0.9069	C17—F18	1.3251 (18)
N8—C9	1.364 (2)	C17—F20	1.332 (2)
N8—H8	0.9582	C17—F19	1.3437 (19)
			
C6—C1—C2	120.18 (15)	C10—C9—C14	118.74 (14)
C6—C1—N8	119.58 (15)	C11—C10—C9	120.30 (13)
C2—C1—N8	120.13 (14)	C11—C10—H10	119.8
N7—C2—C1	120.94 (15)	C9—C10—H10	119.8
N7—C2—C3	121.67 (16)	C10—C11—C12	121.08 (14)
C1—C2—C3	117.36 (17)	C10—C11—H11	119.5
C4—C3—C2	121.20 (19)	C12—C11—H11	119.5
C4—C3—H3	119.4	C11—C12—C13	118.05 (14)
C2—C3—H3	119.4	C11—C12—C15	124.48 (14)
C3—C4—C5	121.06 (18)	C13—C12—C15	117.46 (13)
C3—C4—H4	119.5	C14—C13—C12	121.44 (13)
C5—C4—H4	119.5	C14—C13—H13	119.3
C4—C5—C6	119.0 (2)	C12—C13—H13	119.3
C4—C5—H5	120.5	C13—C14—C9	120.38 (14)
C6—C5—H5	120.5	C13—C14—H14	119.8
C5—C6—C1	121.21 (18)	C9—C14—H14	119.8
C5—C6—H6	119.4	O16—C15—C12	125.88 (14)
C1—C6—H6	119.4	O16—C15—C17	115.33 (14)
C2—N7—H7A	123.8	C12—C15—C17	118.78 (13)
C2—N7—H7B	110.6	F18—C17—F20	107.33 (14)
H7A—N7—H7B	119.4	F18—C17—F19	106.72 (14)
C9—N8—C1	125.23 (12)	F20—C17—F19	107.33 (13)
C9—N8—H8	122.1	F18—C17—C15	111.47 (13)
C1—N8—H8	111.3	F20—C17—C15	112.54 (13)
N8—C9—C10	122.24 (13)	F19—C17—C15	111.16 (13)
N8—C9—C14	119.02 (13)		
			
C6—C1—C2—N7	−176.94 (16)	C10—C11—C12—C13	0.2 (2)
N8—C1—C2—N7	6.8 (2)	C10—C11—C12—C15	179.43 (14)
C6—C1—C2—C3	1.2 (2)	C11—C12—C13—C14	−0.7 (2)
N8—C1—C2—C3	−175.01 (15)	C15—C12—C13—C14	−179.98 (14)
N7—C2—C3—C4	177.89 (19)	C12—C13—C14—C9	0.9 (2)
C1—C2—C3—C4	−0.3 (3)	N8—C9—C14—C13	179.40 (14)
C2—C3—C4—C5	−1.1 (3)	C10—C9—C14—C13	−0.5 (2)
C3—C4—C5—C6	1.4 (3)	C11—C12—C15—O16	−175.65 (16)
C4—C5—C6—C1	−0.4 (3)	C13—C12—C15—O16	3.6 (2)
C2—C1—C6—C5	−0.9 (3)	C11—C12—C15—C17	5.5 (2)
N8—C1—C6—C5	175.34 (17)	C13—C12—C15—C17	−175.33 (14)
C6—C1—N8—C9	107.87 (18)	O16—C15—C17—F18	−1.0 (2)
C2—C1—N8—C9	−75.9 (2)	C12—C15—C17—F18	178.04 (14)
C1—N8—C9—C10	5.3 (2)	O16—C15—C17—F20	119.68 (16)
C1—N8—C9—C14	−174.67 (15)	C12—C15—C17—F20	−61.32 (19)
N8—C9—C10—C11	−179.88 (14)	O16—C15—C17—F19	−119.90 (15)
C14—C9—C10—C11	0.1 (2)	C12—C15—C17—F19	59.11 (19)
C9—C10—C11—C12	0.1 (2)		

### Hydrogen-bond geometry (Å, °)

**Table:**

*D*—H···*A*	*D*—H	H···*A*	*D*···*A*	*D*—H···*A*
N7—H7B···F19^i^	0.91	2.32	3.2077 (19)	166
N8—H8···O16^ii^	0.96	2.06	2.9757 (17)	159

Symmetry codes: (i) −*x*+1, *y*−1/2, −*z*+3/2; (ii) *x*−1/2, −*y*+3/2, −*z*+1.
